# Indents

**Published:** 1903-04-15

**Authors:** 


### INDENTS.

☞ **Brief Articles of Technical or Scientific Value will receive notice and comment. Contributions invited.**

**Sterlizing Broaches.** Cleanse the broaches thoroughly after using, and keep in a small bottle of undiluted Lysol. The alkaline fluid prevents rusting.—G. B. Winter in *Dental Era.*

**Inserting Gutta-percha or Cement.** To keep gutta-percha or cement from adhering to the instrument while inserting in a cavity, dip the instrument into talcum powder or powdered soapstone.—*Dental Review.*

**A Napkin Point.** There is one point perhaps some of the younger men have not had demonstrated to them, insignificant, perhaps, but useful. If it is desired to keep dry a left lower tooth for ten, fifteen, or twenty minutes, act as follows:

Fold or roll a napkin into a strip one and a half inches wide and not less than seven or eight inches long. Have the patient raise the tip of the tongue to the roof of the mouth. Place the end of the napkin against the lingual surfaces of the right lower teeth and pass it across the mouth under the tongue to the lingual surfaces of the left lower teeth. Let the tip of the tongue be depressed. Pass the remainder of the napkin back across the mouth from left to right, this time over the tongue. Stand to the right and in front of your patient. Place a cotton roll between the cheek and left upper teeth to occlude the parotid duct. Hold the tongue firmly down against the floor of the mouth with the fingers of the left hand, the thumb being under the chin. The lower fold of napkin occludes the submaxillary and sublingual ducts and the upper fold enables the operator to hold the tongue down without it slipping. The whole secret of success lies in keeping the tongue firmly down against the floor of the mouth. The patient may at first makes unconscious spasmodic efforts to raise the tongue to swallow, but if it is held in place these efforts will cease.

For keeping dry the right lower teeth, reverse the operation.—G. E. Hunt in *Dental Brief*.

**Fusing Points of Porcelain Bodies.**

Dr. W. A. Capon, in the March *Dental Brief*, gives the following as the result of a research made to determine the exact fusing points of the various porcelain bodies on the market. He determined the temperature by a pyrometer made by Kaiser and Schmidt, of Berlin, and of certified accuracy:

| | Temperature. |
|---|---|
| Downies | 1544 |
| Jenkins's | 1544 |
| Ash's low fusing | 1544 |
| Ash's high fusing | 1904 |
| Moffitt's porcelain | 2047 |
| Brewster's enamel | 2084 |
| Consolidated high fusing | 2192 |
| Brewster's foundation body | 2210 |
| Whiteley's porcelain | 2210 |
| Close's foundation body | 2300 |
| White's porcelain (inlay) | 2300 |
| Parker's body | 2586 |
| Ash & Son's tooth body | 2264 |
| Sibley's tooth body | 2408 |
| Dental Protective tooth body | 2440 |
| Justi's tooth body | 2440 |
| S. S. White's tooth body | 2516 |
| Johnson & Lund's tooth body | 2586 |
| Lukens's tooth body | 2606 |
| Century tooth body | 2604 |
| Consolidated Dental Manufacturing Company's tooth body | 2624 |

For three reasons these temperatures can be accepted as being absolutely correct, for every detail was given the most minute attention. First, the voltage was controlled to 110 without deviation. Secondly, these figures represent several trials of the same material, and, thirdly, the watch was assisted by the eye, which is the only sure way of knowing when porcelain is properly fused.

**Should Colleges Educate Scientists and Specialists.** The first and the chief concern of a dental school, it seems to me, should be to give the student a thorough knowledge of all that a useful dentist is required to know. Above and beyond this he should know enough of the collateral sciences to keep in touch with professional men, and to take advantage of such aid as these sciences may afford in solving problems that overreach the bounds of his professional bailiwick. The more profound delving into the sciences and branching into specialties should be an elective post-graduate course. The dental colleges cannot make any notable percent of their students Underwoods or Millers nor yet oral surgeons or expert crown and bridge-workers. It should not be attempted, because the world needs a very much greater number of good all-round workmen than it does experts in any one line, and the colleges should plan to produce these. When they have produced a good all-round workman they have produced a good, sturdy, healthy stock upon which anything else can be readily and successfully grafted.

A specialist who becomes one during his college course usually lacks that broad comprehensive knowledge of his profession upon which his usefulness most depends. While, on the other hand, a specialist who becomes one by following his inclination or opportunity, and a scientist who finds in science his hobby, are usually useful and successful men.—Dr. W. H. Truman in *Dental Brief*.

**Pulpless Teeth and Neurosis.** The best men in the profession—the ones who have made American dentistry respected the world over—have been led to place a great value on the life and preservation of every tooth in the head. That has been carried to such an extent that men have often said that no tooth should ever be sacificed or lost, and it is a point of warning that I would echo very strongly. The teeth have a very great value in the human economy—no one appreciates that more than we as dentists; but as dentists practicing a specialty of medicine we are all in danger at times of overvaluing that part of the body on which we happen to be operating, and this word of warning that we should not value any part of the dental economy where the life of the body itself is at stake is not only timely, but a very valuable caution to the very best dentists—those men who are a pride to the profession and to whom it is one of the greatest pains to be compelled to authorize the loss of one of the natural teeth.

In the regard to the other point of Dr. Hart—the difficulty of keeping a pulpless tooth in a condition free from irritation —I take decided exception to his line of argument. I do not believe that the paper this evening can be made to cover any such field as that. During the twenty odd years that I have been engaged in practice I have seen a number of cases of this kind, and I have yet to see demonstrated a case where the neurosis can be directly attributed to a pulpless tooth on account of any purulence in the canal, if the canal has been properly filled. I believe, furthermore, that there is no reason why all canals, with a very limited exception, cannot be first properly cleaned and then filled. It would take too long to go into that question in detail; the point I wish to make is that while there may be a very limited percentage of teeth in which this is impossible, those are the teeth that may have to be sacrificed.—Dr. Rhein in *Cosmos*.

**The Intravenous Injection of Formalin.** Dr. C. C. Barrows, of New York, very recently had under treatment a case of puerperal septicemia of such severity as to lead to the belief that a fatal termination was rapidly impending. As a last resort he ordered the intravenous injection of a 1-5000 solution of formalin in sterile normal saline solution. The exceedingly high temperature of the patient was rapidly lowered, and the examination of the blood soon after showed that the streptococci had disapeared. Since this first case several persons suffering in a similar manner have been subjected to the same treatment with apparently good effect. It is indeed too early to say whether or not Dr. Barrows' experiment is one that is destined to assume an importance commensurate with the apparent value of the results so far achieved. The efficiency of formaldehyde as an antiseptic is thoroughly recognized, and we know that certain drugs, such, for instances, as urotropin, owe their antiseptic action to a decomposition resulting in the liberation of formaldehyde. In one instance since the first case was reported, another surgeon by mistake employed a 1-2500 solution, with nearly fatal results, as the patient suffered immediately from an attack of asphyxia, during which her life was for some time in jeopardy. In the strength recommended by Dr. Barrows, however, the solution seems to be quite harmless, and will undoubtedly be extensively tried within a short time, so that a few weeks may suffice to reveal the true value of what would seem to be an important discovery. The therapeutic utility of intravenous medication forms a most important subject for the present generation. Are we destined to be able to directly sterilize the blood stream of living beings? Is it likely that we shall ever be able to rapidly alter the blood's proportion of hemoglobin in severe forms of anemia, by introducing into the circulation some preparation of iron that will at once be taken up by the red corpuscles? May we expect that substances will be found that will rapidly and safely modify the condition of an exhausted myocardium, or increase a phagocytosis, or promote trophic phenomena? These are problems still too much neglected by physiological chemistry, and the solution of which might bring about profound changes in our therapeutic methods. At the present moment the importance of Dr. Barrows' observations lies chiefly in the fact that they relate to a subject still very obscure, but on which the near future is destined to throw much light.—Editorial in *International Journal of Surgery*.

